# Resilience and Job Satisfaction: Effect of Moderated Mediation on the Influence of interpersonal Justice on the Performance of Public Servants

**DOI:** 10.3390/ijerph20042957

**Published:** 2023-02-08

**Authors:** Jazael Albalá-Genol, Pedro Antonio Díaz-Fúnez, Miguel Ángel Mañas-Rodríguez

**Affiliations:** 1 IPTORA Research Group, 04120 Almería, Spain; 2 Psychology, University of Almería, 04120 Almería, Spain

**Keywords:** resilience, job satisfaction, interpersonal justice, intra-role performance, job demands-resources model

## Abstract

The perception of interpersonal justice is one of the key resources for improving employees’ performance intention. Elements such as employees’ level of satisfaction or their self-perception of their ability to cope with problematic situations are key factors in this relationship according to the job demands-resources model. The objective of this study was to analyze how the perception of job satisfaction and the self-perception of resilience influence how interpersonal justice affects employee performance. A total of 315 public sector employees, who perform administrative and customer service tasks, have contributed to this study. The results show that the relationship between interpersonal justice and intra-role performance is completely mediated by job satisfaction; however, when we include the modulating effect of resilience between interpersonal justice and job satisfaction, the influence of the former is reduced as the self-perception of resilience. This indicates that the positive effects of justice are reduced as workers’ self-perception of resilience increases.

## 1. Introduction

The relationship between job satisfaction and job performance has fascinated researchers for decades, becoming the “Holy Grail” of industrial psychologists [1]. Multiple studies have tried to explain this relationship scientifically [2,3,4,5,6], and even today remains it a constantly investigated relationship [7]. Most of these studies have demonstrated the positive influence of job satisfaction on performance [8,9]. Therefore, interest is focused on more complex models to analyze workplace factors that can influence this relationship.

Two key variables in this line are interpersonal justice and resilience. Authors such as Bakhshi et al. [10], Thomas and Nagalingappa [11], and López-Cabarcos et al. [12] affirm that employees who perceive fair treatment develop higher levels of job satisfaction. Several authors identify resilience as a positive element influencing other variables [13,14,15,16,17,18]. The previous study by Ahmad et al. [19] shows that resilience is a dimension that acts as a mediating variable between other variables in organizations. It seems, therefore, that a high level of resilience in employees would mean a significant increase in their job satisfaction.

However, some theories incite us to doubt the positive effect of resilience in a negative workplace. For example, the Conservation of Resources Theory [20] proposes that if employees were in a contrary work environment with hindrance demands (e.g., low interpersonal justice), people with high resilience could be perceived as a lost resource when it comes to increasing job satisfaction. As a result of the previous situation, workers can think that the organization does not have a suitable environment for their individual development, and their job satisfaction could be reduced.

The current study was carried out in the complex work context of the public sector. Mañas-Rodriguez et al. [21] argued that the public sector is usually subject to some relevant problems: the connection with political authorities; the existence of values that are difficult to reconcile; a bureaucratic functioning that hinders positive emotional bonds; and the need to make unpopular decisions based on general policies. Compared to private organizations, public organization is usually perceived as rigid, slow, and inefficient [22]. Considering all these circumstances, the role of resilience in public employees is a relevant study goal to improve the public sector by considering their resources [23].

For all these reasons, the objective of this research is to analyze how resilience moderates the influence of interpersonal justice on job satisfaction, and the latter on public servants’ intra-role performance. The result of this analysis can be interesting for public administrations since, knowing these results, they could formulate certain organizational strategies to improve employee performance.

The novelty element of our article is to provide a new perspective on resilience. Until now, studies on this variable had only investigated its positive influence on certain variables [13,16]; however, our study proposes a new vision of resilience, providing possible negative effects in the face of certain variables and circumstances of the work context.

## 2. Literature Review, Theory and Research Hypotheses

The job demands-resources theory (JD-R) is one of the most widely used to study psychosocial variables in the work context [24]. From this theory, interpersonal justice is a valuable resource from the work context. Resilience can be considered a personal resource capable of modulating the specific responses of the employee in the context of it. Job satisfaction is an emotional response of the worker, given by the context and mediated by the personal resources available to the person. Finally, intra-role performance is the behavioral intention that derives from the resources available in the work context through that emotional response.

The great interest in studying intra-role performance is that it is necessary to understand the keys of this dimension to improve it since the quality of the product and the achievement of organizational objectives will depend on it. Several authors consider that the success of an organization depends on the good performance of its employees [25]. Therefore, organizations will need to have people with a high level of performance in their teams. Intra-role performance is a dimension of job performance that we can define as those activities that contribute directly or indirectly to the technical base of the organization and these vary in different works within the same organization [26].

In recent years, multiple variables that positively affect employee job performance have been studied. However, one of the most important and most analyzed by many authors is job satisfaction. The relationship between job satisfaction and job performance has been the subject of study by hundreds of authors, including three highly relevant meta-analyses [27,28,29].

Judge and Klinger [30] consider job satisfaction the most researched subject in the history of organizational psychology. Maslow [31] wrote in his Hierarchy of Needs Theory about the job satisfaction of individuals and linked the increase in job satisfaction with the increase in resources obtained through work. Currently, this variable has become so important due to the transcendence of labor policies and to the role that workers are assuming as key pieces for the development of organizational activities [32]. In fact, there are recent studies that positively link job satisfaction with organizational behavior [33].

Recent meta-analyses [34,35] have Locke’s [4] job satisfaction as a reference; conceptualization of this construct as a positive emotional state obtained through our self-perceived experience in the workplace. Mueller and McCloskey [36] defined job satisfaction as “the degree of positive affective orientation toward employment”. Newstrom and Davis [37] considered it as a set of favorable or unfavorable feelings and emotions with which employees view their work, and Robbins [38] defined job satisfaction as a general attitude of the individual towards one’s job. A person with high job satisfaction has a positive attitude, while someone dissatisfied harbors a negative attitude. There are many concepts to define job satisfaction but as we can see, almost all of them refer to the relationship of individuals with the work situation they are in. These definitions of job satisfaction about emotions, feelings, and perceptions in the work environment give us the idea that satisfaction is influenced by the perception of different types of situations at work, such as organizational justice.

Justice has always been an element concerning society throughout its history. In the workplace, it has also been a concept that has always been pursued, as we have seen in different historical periods, such as in the industrial revolution when people already held meetings and tried to match the leader-subordinate relationships to be as fair as possible. In this way, organizations are currently trying to integrate this justice into their structure, thus changing roles. Now it is the organization that has the most interest in integrating this justice into their way of working. Specifically, researchers show more and more interest in studying interactional justice, since it is the type of justice that most directly affects the quality of the leader-subordinate relationship [39]. The great interest in studying interpersonal justice is because it acts within the organization as an interpersonal resource that strengthens employees’ connection to their work and to the people they work with [40].

Bies and Moag [41] had already introduced the concept of interactional justice in previous studies, remarking that interactional justice was primarily concerned with how people interact and their perception of justice, and emphasizing that the employee’s perception of justice in the process of procedure execution would be influenced by the attitude of the executors towards them and how the executors treated them. Bradley and Sparks [42] defined interactional justice as the attitudes and behaviors adopted by the individuals in the relationship. Ando and Matsuda [43] assumed that the concept of interpersonal justice was formed by employees’ feelings about how they were treated during the process of the execution of the procedure. Cropanzano and Greenberg [44] defined interpersonal justice as the degree to which people feel when treated with courtesy, dignity, and respect by their immediate or superior leaders.

Many studies affirm the existence of the influence of interpersonal justice on job satisfaction. Bakhshi et al. [10] pointed out that those who perceived that there was justice in their organizations were more likely to be satisfied with their work. In Saudi Arabian manufacturing employees, interpersonal justice had positive effects on job satisfaction [45]. The empirical study by Thomas and Nagalingappa [11] on professionals from different fields shows the positive impact of interpersonal justice on job satisfaction. Interpersonal justice had significant positive correlations with job satisfaction among Nigerian university professors [46].

Resilience is an element that companies have studied in recent years due to its strong impact on workers and its influence on other variables existing in the work environment [47,48]. That is why we have found it interesting to study how this variable modulates the influence between two variables.

Resilience, seen from an organizational point of view, can be defined as a complex combination made up of interactions, behaviors, and perspectives [49]. More generally, resilience has been defined by many authors in recent years. For Luthar et al. [50], resilience refers to a dynamic process that encompasses positive adaptation within the context of significant adversity, while Masten [51] defines it as a strategy of positive adaptation to adversity despite serious threats to adaptation or development. The American Psychological Association [52] offers a broader concept of the term resilience, defining it as the process of adapting well in the face of adversity, trauma, tragedy, threats, or even significant stressors, such as family and relationship problems, health problems, as well as situations that generate stress in the workplace or with the financial environment. The development of resilience is not strictly limited to the person’s recovery but is a resource that allows them to prosper in a better position than the state of balance that they were in before [53]. Previous research found that employee resilience can be improved through human resource processes [19,54,55].

Several authors affirm the positive influence of resilience in other work areas. In research by Youssef and Luthans [17], the results showed that resilience significantly and positively influenced job satisfaction, commitment, and happiness at work. The study by Liossis et al. [14] revealed that with the development of resilience through specific training programs in adults, positive effects on the well-being of people were obtained, both personally and at work. Reivich and Schatte [16] suggested that resilience can assume a key role in unemployed people, since it will help them overcome the adversities derived from unemployment, thus favoring their reincorporation into the labor market. Pereira et al. [56] suggest that people who are part of resilient organizations generate positive economic dynamism, which reinforces the company’s human capital and makes it a competitive advantage.

However, not all perspectives identify resources as positive elements. Hobfoll [20] formulated a theory known as the Theory of Conservation of Resources, which suggested that people tend to retain, protect, and build resources, and what is threatening is the potential or actual loss of these valuable resources. According to this theory, resources can be transformed into negative elements when people assume investments in the development of their capacities as lost, not achieving the expected returns [57]. Thus, it is assumed that resources are beneficial when they generate other resources or reduce demands, but if this does not happen, resources could assume a negative role [58]. Dressier’s study [59] affirmed that in certain situations, people can perceive a resource as negative, with negative consequences for their satisfaction if they consider that it is not useful for them to achieve more relevant objectives. In this way, high levels of perception of resilience could be expected to have negative effects in the workplace context. Employees who perceive their resilience capacity highly, when developing their activity in a work context with high levels of interpersonal justice, may perceive the non-use of this resource as a loss of their skills, which will make them feel less satisfied at work. This could cause the high levels in which the employees perceive their resilience to negatively affect the influence of interpersonal justice on job satisfaction.

The hypotheses proposed for this study are the following:

**Hypothesis** **1.** *Interpersonal justice will show a significant positive influence on resilience and job satisfaction.*

**Hypothesis** **2.** *The effect of interpersonal justice on job satisfaction is moderated by the level of employee resilience.*

**Hypothesis** **3.** *The degree of interpersonal justice will influence the improvement of the employee’s intra-role performance, through job satisfaction. Although in line with the moderate mediation model [60], this mediation will be subject to the level of employee resilience.*

This research tries to study a new perspective on the influence of resilience in the work context. Until now, studies have focused on a positive perspective of this variable, not finding results in which it has a negative or detrimental influence. The present study tries to explore this option, which would offer a new vision of resilience, not only as a positive element in all contexts but also as a variable that must be studied and adapted to each contextual situation.

## 3. Materials and Methods

### 3.1. Process and Sample

The data were collected from a Spanish public organization. The researchers contacted the public administration management and explained the objective of their project. After accepting the proposal, the organization’s management set out to inform employees about the importance of the study to be carried out, commenting on the confidentiality and anonymity of the questionnaires, thus ensuring maximum employee participation. All members of the study population (public university administration and service workers) were invited to participate. The authors collected the data from public workers. The authors collected the data through Google Forms. This process was carried out using this methodology because it is one of the most effective and safe ways to do it. An example of this method can be seen in the recent study by Hussain et al. [61]. In the present work, a total of 380 responses were obtained. After removing incomplete responses and wrong answers, a total sample of 315 workers was finally obtained (response rate = 82.89%).

The sample is distributed according to the variables shown below. Regarding the gender variable, the sample was composed of 51.6% men and 48.4% women. In the age variable, 1.8% of subjects were under 36 years old, 14.1% were between 36 and 45 years old, 64% were between 46 and 55 years old and 20.1% of individuals were over 55 years old. In relation to academic level, 10.3% had finished elementary or middle education; 23% were educated to a high school level; 55.5% had a bachelor’s degree and 11.2% had completed postgraduate or doctoral studies. Regarding employment status, 92.9% were public officials and 7.1% were ordinary public servants. The different jobs were arranged as follows: 7.1% were managers; 15.9% were pre-managers and 77% were regular workers. An hourly distribution was assigned so that 81.9% worked in the morning shift and 18.1% in the afternoon shift.

### 3.2. Instrument

Intra-role performance has been measured using the Goodman and Svyantek [26] performance scale. This scale is made up of three items (i.e., the employees of my work group achieve the objectives of the work.). The response format for the items was a 7-point Likert type, ranging from 1: “Totally disagree” to 7: “Totally agree”.

To measure job satisfaction, the job satisfaction scale of Warr et al. [62] has been used. This scale consists of 9 items (i.e., indicate to what extent you are satisfied with your peers.). The type of response given for all items presents a 7-point Likert-type format, from 1: “Totally dissatisfied” to 7: “Totally satisfied”.

We used the Colquitt [63] organizational justice questionnaire to measure interpersonal justice. This questionnaire uses 7 items (i.e., to what extent have you refrained from making inappropriate comments or observations?). The answers to this questionnaire are 5-point Likert type, ranging from 1: “Strongly disagree” to “5: Strongly agree”.

Resilience was measured through the PCQ-12 psychological capital questionnaire [64]. This consists of three items measuring resilience. (i.e., I can cope well with difficult times at work because I have experience in overcoming difficulties). The responses to this questionnaire present a 6-point Likert-type format, from 1: “Totally disagree” to 6: “Totally agree”.

### 3.3. Analysis 

**Figure 1:**
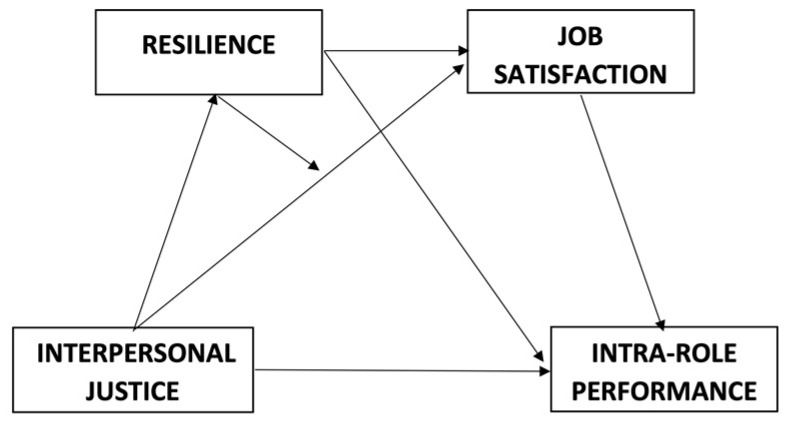
Research Model.

All the data were analyzed with SPSS 25. After calculating descriptive data, Cronbach’s alphas were performed as well as the zero-order relationships between all constructs, mediation, and moderation analyses. Figure 1 shows the conceptual model tested in the current study. As in Cristea et al. [65], a multiple mediation analysis was tested where the effect of interpersonal justice on intra-role performance is mediated by job satisfaction, whose effect is further extended by reducing the level of satisfaction perceived by employees, which is one of the performance antecedents. Furthermore, the model also includes resilience as a moderator of the relationship between interpersonal justice and job satisfaction. Mediation and moderation analyses were performed with the non-parametric starting procedure to estimate direct and indirect effects using the PROCESS package [59]. As this specific conceptual model is not covered in the process, we followed Hayes’ suggestion and we first performed the multi-step mediation analysis of the effect of interpersonal justice on intra-role performance, with resilience and job satisfaction as mediators 1 and 2, respectively (Model 6 in PROCESS). Second, a simple moderation analysis was carried out to analyze whether resilience would moderate the relationship between interpersonal justice, job satisfaction, and intra-role performance (Model 7 in PROCESS).

Indirect and conditional effects were considered significant if the 95% bootstrapping confidence intervals (CI) for those effects were based on 10,000 baseline samples which did not include zero. Mediation effect size was calculated using the fully standardized indirect effect (abcs) [66] and providing bootstrapping confidence intervals of 95% BC. This measure of effect size is based on the product of betas for routes “a” and “b”, and can be interpreted as the expected change in the dependent variable (i.e., Intra-role performance) per unit change in prediction variables (i.e., interpersonal justice) that occurs indirectly through the mediator (i.e., job satisfaction). Finally, the Johnson-Neyman technique [67] was used to derive the moderator value (i.e., resilience score), in which the effect of the predictor variable (i.e., interpersonal justice score) transits between statistically significant and non-significant at an alpha level of 0.05.

## 4. Results

Table 1 shows the descriptive data, the internal consistencies obtained for each scale, and the correlations between the measures of the present study. Participants’ mean scores on interpersonal justice, job satisfaction, resilience, and intra-role performance were above the centered scale point. Internal consistencies of the scales ranged from 0.81 (resilience) to 0.91 (intra-role performance) and, using these instruments, they were like previous evidence. As expected, all constructs were strongly correlated.

Testing the mediation model, a multiple mediation analysis of the relationship between interpersonal justice and intra-role performance was carried out, with resilience as Mediator 1 and job satisfaction as Mediator 2 (see Figure 1). Table 2 shows the results of the models tested in this mediation analysis. In the first model, interpersonal justice was a significant predictor of resilience (Mediator 1). The second model shows that both interpersonal justice and resilience were significant predictors of Mediator 2 (job satisfaction). According to the third model, the total effect of interpersonal justice on intra-role performance was significant (TE = 0.245, SE = 0.060, *p* < 0.001). Finally, the fourth model shows that all the predictive variables were a significant predictor of intra-role performance when all their variables were included in the regression analysis. Compared to the previous model, the interpersonal justice coefficient decreased from 0.245 to −0.036.

Table 3 presents the general and specific indirect effects (IE). The results showed significant mediation, with a total IE of 0.281 (SE = 0.051, 95% CI BC from 0.191 to 0.389) and a very large effect size (abcs = 0.257, 95% CI BC 0.184 to 0.337). All specific indirect effects were also significant (IE1: TE = 0.053, SE = 0.024, 95% CI BC from 0.015 to 0.107, abcs = 0.048; IE2: TE = 0.198, SE = 0.035, 95% CI BC from 0.132 to 0.271, abcs = 0.181; IE3: TE = 0.030, SE = 0.013, 95% CI BC from 0.009 to 0.059, abcs = 0.028).

Table 4 shows that moderation analysis revealed that the effect of interpersonal justice on job satisfaction was influenced by the level of employee resilience (interaction coefficient: interpersonal justice x resilience = −0.113, SE = 0.053, *p* < 0.05). Specifically, as also shown in Table 4, the Johnson-Neyman technique showed that the conditional effect of interpersonal justice on job satisfaction was significant in all resilience scores with the smallest error level in the range of 5.75–6.00.

## 5. Discussion

The objective of this study is to analyze the moderating effect that resilience has on the influence of interpersonal justice on job satisfaction and, consequently, on workers’ intra-role performance. Observing the results obtained, it can be confirmed that the hypotheses have been supported. In the first hypothesis, the results show a positive influence of interpersonal justice on resilience and job satisfaction. The second hypothesis is confirmed by proving that resilience negatively moderated the positive influence of interpersonal justice on job satisfaction. Our third and last hypothesis, the moderated mediation model, is demonstrated. The level of employee resilience affects the relationship of interpersonal justice in intra-role performance through its influence on employee job satisfaction.

Our results are supported by previous studies of Bakhshi et al. [10], Thomas and Nagalingappa [11], and López-Cabarcos et al. [12], which showed the positive influence of justice on job satisfaction. However, the results show that the positive influence of interpersonal justice on job satisfaction is negatively moderated by resilience. This result is in line with the Conservation of Resources Theory [20], where employees, in a context of high interpersonal justice, will try to protect and improve the usefulness of their resilience, thus reducing their job satisfaction. These results support the existence of “negative spirals” of losses [20,68], according to which the negative effect of resilience on the positive influence of interpersonal justice on job satisfaction would cause it to decrease and in turn the decrease in job satisfaction would also cause the level of employees’ job performance to decrease.

### 5.1. Theoretical Implications

The present study has three theoretical implications in its contributions. First, it proposes the ability to negatively influence job resources. In the job demands-resources model [24], job resources only positively influence the organizational context, either to cope with job demands or to generate resources. The studies carried out to date also consider resilience as a personal resource of employees, with a positive influence in the work context [14,16,17]. However, our study provides a new perspective on resilience, according to which it can have negative consequences for employees under certain organizational circumstances.

Second, our results support the Conservation of Resources Theory [20]. This theory proposes that the possession of a resource such as resilience can be negatively valued. Our results suggest that a high level of worker resilience may lead to a reduction in the effect of job satisfaction. In our results, as the level of resilience of employees increases, the positive impact of interpersonal justice on job satisfaction decreases, which implies a reduction of the latter and in turn of intra-role performance. When public workers have high levels of resilience, their institution must protect them, considering the adverse consequences that a context of high interpersonal justice will have on their performance.

The third theoretical implication is on the JD-R theory [24]. Our results add an amplified view of the interaction between job resources in this theory. Following the JD-R model, it is essential to understand how organizational context resources (i.e., interpersonal justice) and employee resources (i.e., resilience) interact. A context where there is no coherence between existing resources can turn positive characteristics into a negative element on the performance of the employees.

### 5.2. Practical Implications

This paper presents three relevant practical implications. The first is the importance of making the work context and the employee compatible. Our results show that public employees must be in work contexts consistent with their work resources to achieve their best performance. Otherwise, the effects can be inadequate for this employee and the institution.

The second practical implication refers to the personnel selection process. Choosing employees with personal resources that are not adequate for the context in which they are going to work can hurt them and the organization. Therefore, public entities must avoid hiring the most qualified candidate, but rather the most appropriate to the context of their day-to-day activities.

The third practical implication of this work is oriented to the control of demands in organizational contexts. Public administrations must create environments with a controlled number of demands. These could act as challenges for the employees if they have been planned according to the personal resources that the worker presents. This situation will increase the satisfaction of public employees and their performance.

### 5.3. Limitations and Future Research

Similar to other studies, ours has a series of limitations which are necessary to inform for possible future research. Regarding limitations, we used online questionnaires to extract the information, which could suppose that these were affected by the variance of the common method [69]. For future research, it would be recommended to introduce, in the measurement method, types of variables that imply an intersubjective response at the team level by the employee. The use of instruments such as interviews, direct observation, or the collection of objective data on productivity and performance could add more validity to the data collected. Another limitation of our study is found in the analyzed sample; as our analysis focuses on public employees in Spain, it is considered a very specific sample and difficult to compare to other types of organizations. In future research, it would be interesting to compare different samples taken from public and private administrations [23,70], as well as to study organizations from different public bodies (state, autonomous and local) or to carry out cross-cultural studies. Finally, our research provides less information than others due to the cross-sectional nature of its design, so it would be necessary to carry out multilevel and longitudinal studies, which would make it possible to analyze the effects of group membership and, in turn, the analysis of the evolution of variables to improve behaviors, attitudes and group performance of work teams, or to study how these variables influence performance and productivity and, indirectly, favorable organizational behaviors such as cordiality or civility.

## 6. Conclusions

In conclusion, our study offers a new approach to employee resources in the workplace, showing that they can have a negative influence on job satisfaction and on their intention to strive for performance. Thus, the accumulation of resources in an organizational context, without a utility or adequate use, will lead to negative emotional states and a consequent reduction in the level of intra-role performance. Another implication is that employees with a high level of resilience do not need interpersonal justice to feel job satisfaction.

## Figures and Tables

**Table 1:** Descriptive data, internal consistencies, and correlations.

	M	SD	α	2	3	4
1. Interpersonal justice	3.72	0.96	0.84	0.476 **	0.178 **	0.224 **
2. Job satisfaction	5.22	1.11	0.90		0.388 **	0.538 **
3. Resilience	4.63	0.87	0.81			0.434 **
4. Intra-role performance	4.86	1.05	0.91			

Note: M = Mean; SD = Standard Deviation; α: Cronbach’s alpha; ** *p* < 0.001.

**Table 2:** Results from regression analyzes examining the mediator model of resilience (M1) and job satisfaction (M2) in the effect of interpersonal justice on intra-role performance.

	Coefficient	SE	*p*
Model 1 (Resilience)			
X (Interpersonal justice)	0.171	0.051	<0.001
Constant	3.996	0.195	<0.001
R^2^ = 0.035			
F = 11.349, *p* ≤ 0.001			
Model 2 (Job satisfaction)			
X (Interpersonal justice)	0.469	0.055	<0.001
M1 (Resilience)	0.419	0.060	<0.001
Constant	1.517	0.318	<0.001
R^2^ = 0.322			
F = 74.147, *p* ≤ 0.001			
Model 3 (Intra-role performance) Total Effect			
X (Interpersonal justice)	0.245	0.060	<0.001
Constant	3.946	0.231	<0.001
R^2^ = 0.050			
F = 16.544, *p* ≤ 0.001			
Model 4 (Intra-role performance)			
X (Interpersonal justice)	−0.036	0.057	<0.001
M1 (Resilience)	0.309	0.060	<0.001
M2 (Job satisfaction)	0.422	0.052	<0.001
Constant	1.363	0.305	<0.001
R^2^ = 0.345			
F = 54.694, *p* ≤ 0.001			

**Table 3:** Indirect effects of the serial multiple mediator model of the effect of interpersonal justice (X) on intra-role performance (Y) through resilience (M1) and job satisfaction (M2).

			Bootstrapping BC 95% CI	
	Coefficient	SE	Lower	Upper
Overall indirect effect	0.281	0.051	0.191	0.389
IE1:X => M1 => Y	0.053	0.024	0.015	0.107
IE2:X => M2 => Y	0.198	0.035	0.132	0.271
IE3: X => M1 => M2 => Y	0.030	0.013	0.009	0.059

**Table 4:** Results of the regression analysis examining the modulating effect of resilience on the influence of interpersonal justice on job satisfaction.

Antecedent	Coefficient	SE	*p*
X (Interpersonal justice)	0.995	0.253	<0.001
W(Resilience)	0.830	0.202	<0.001
XxW	−0.113	0.053	0.034
Constant	−0.378	0.946	0.690
R^2^ = 0.332 F = 51.494, *p* = 0.000			
Johnson-Neyman Analysis			
Resilience Scores	Coefficient	SE	T
1.00	0.882	0.202	4.369 **
1.25	0.854	0.189	4.515 **
1.50	0.826	0.176	4.679 **
1.75	0.797	0.164	4.865 **
2.00	0.769	0.151	5.079 **
2.25	0.741	0.139	5.325 **
2.50	0.713	0.127	5.610 **
2.75	0.685	0.115	5.941 **
3.00	0.656	0.103	6.327 **
3.25	0.628	0.093	6.774 **
3.50	0.600	0.082	7.282 **
3.75	0.572	0.073	7.831 **
4.00	0.543	0.065	8.362 **
4.25	0.515	0.059	8.746 **
4.50	0.487	0.055	8.790 **
4.75	0.459	0.055	8.347 **
5.00	0.431	0.058	7.467 **
5.25	0.402	0.063	6.377 **
5.50	0.374	0.071	5.297 **
5.75	0.346	0.080	4.341 **
6.00	0.318	0.090	3.539 **

Note: ** *p* < 0.001.

## Data Availability

The data presented in this study are available upon request from the corresponding author. The data is not publicly available because it contains private data of the study subjects.
